# Fast sintering of silver nanoparticle and flake layers by infrared module assistance in large area roll-to-roll gravure printing system

**DOI:** 10.1038/srep34470

**Published:** 2016-10-07

**Authors:** Janghoon Park, Hyi Jae Kang, Kee-Hyun Shin, Hyunkyoo Kang

**Affiliations:** 1 Department of Mechanical Design and Production Engineering, Konkuk University, Seoul, Korea; 2 Flexible Display Roll-to-Roll Research Center, Konkuk University, Seoul, Korea; 3 Digital Printing and Imaging Technology, Technische Universität Chemnitz, Chemnitz, Germany

## Abstract

We present fast sintering for silver (Ag) nanoparticle (NP) and flake layers printed using roll-to-roll (R2R) gravure printing. An infrared (IR) sintering module was applied to an R2R system to shorten the sintering duration of an R2R gravure-printed Ag layer. IR sintering of the conductive layer was improved by optimising the process condition. After printing of the Ag NP and Ag flake layers, additional IR sintering was performed in the R2R system. The lowest sheet resistance obtained in the Ag NP layer was 0.294 Ω/□, the distance between the substrate and lamp was 50-mm long, the IR lamp power was 500 W, and the sintering time was 5.4 s. The fastest sintering of 0.34 Ω/□ was achieved with 50-mm distance, 1,000-W IR lamp power, and 1.08-s sintering time. In the Ag flake layer, the lowest sheet resistance obtained was 0.288 Ω/□ with a 20-mm distance, 1,000-W IR lamp power, and 10.8-s sintering time. Meanwhile, the fastest sintering was obtained with a 3.83 Ω/□ sheet resistance, 20-mm distance, 1000-W IR lamp, and 1.08-s sintering time. Thus, the IR sintering module can easily be employed in an R2R system to obtain excellent layer sheet resistance.

Printed electronic devices with excellent electrical performance, flexibility, low cost, and light weight are required in many research fields^1^. Thus, flexible films produced by roll-to-roll (R2R) printing and fast sintering of printed materials have been studied for various applications based on conductive electrodes, such as organic photovoltaic light, organic light emitting diodes, flexible printed circuits, thin-film transistors, radio-frequency identification tags, and sensors^2^,^3^,^4^,^5^,^6^,^7^. Various studies attempted to employ a high conductive layer using different sintering methods, specifically either chemical sintering or heat-treatment-based sintering^1^. Chemical sintering is simple and easy to perform by tuning the conductive ink, which is designed for the self-sintering mechanism of the reaction between a silver (Ag) nanoparticle (NP) and a destabilising agent^8^. In addition to the material-tuning method, temperature-based Ag-sintering techniques have been investigated, including thermal^9^, electrical^10^, plasma^11^, infrared (IR)^12^, ultraviolet (UV)^13^, laser^14^, intense pulsed light (IPL)^15^, and microwave^16^ approaches. (The details and research results were reported by Wünscher *et al*.^1^).

Thermal sintering can typically be performed using a hot-air oven or furnace equipment. This method is widely used for R2R continuous printing systems as an online module or operated as individual equipment for independent sintering. The performance of the thermal-sintering process, including a 150 °C drying temperature with a 5-m-long dry chamber, and that of the sintering furnace equipment at 400 °C for 20 min and 16 μΩ·cm of resistivity have been reported^9^. However, the use of thermal-sintering technique prevents the employment of high temperature in the R2R inline process because of the instability of polymer-based flexible substrate up to the glass-transition temperature. Therefore, the sintering duration should be increased to sinter the conductive layer while controlling the temperature.

Electrical sintering is a cost-effective method of constructing a sintering module. It is selective, effective, and fast without causing substrate damage. Using electrical sintering, Allen *et al*. reported a 120 mΩ/□ sheet resistance with 50-W power and 25-mm/s processing speed^10^. Nevertheless, it was not compatible with the R2R process due to the abstruse contact with moving electrical layers. In addition, plasma sintering based on gas treatment at low pressure is scientifically important. Wolf *et al*. presented resistivity of 3.98-μΩ·cm by applying 300-W power using argon gas for 60 min. Nevertheless, plasma sintering cannot be applied to the R2R process because of the attendant atmospheric pressure process for the substrate drive^11^. Moreover, it requires sintering time and pressure condition that are aligned with the vacuum process.

The various photonic sintering approaches can be categorised as IR, UV, laser, and IPL according to the specific wavelength ranges of the lighting sources^1^. The IR method is very suitable for the R2R process. It can rapidly sinter an Ag layer. Moreover, it can be inexpensively installed in R2R systems. However, IR can damage the flexible substrate or printed layers if the IR conditions are not properly tuned. Thus, the sintering condition of the IR process should be instantly optimised by determining the power of the IR lamp, distance between the IR lamp and substrate, and sintering time. In a previous study, a 2,600 °C IR lamp filament temperature was applied for 1.25 s, and a sheet resistance of 260 mΩ/□ was obtained in an inkjet printing method using patterns^12^.

UV sintering was reported with excellent electrical performance close to 18–43% of the bulk conductivity (2.7·10^7^ S/m) of Ag under 70-mW/cm^2^ power and 100 °C temperature, combined with additional thermal-sintering for 30 min^13^. This method is cost-effective compared with the other methods that employ more expensive equipment (e.g. the IPL machine of NovaCentrix Co. Ltd., USA). However, its usage is limited with UV-reactive materials. Additionally, its long sintering time makes the UV technique incompatible with a continuous R2R process.

The laser-sintering technique has been the focus in many electronics application fields owing to its controllability with small spots (selectivity to patterns) and because it causes less damage. Laser sintering has the advantage of precise controllability, which was reported to have a sheet resistance of 1 Ω/□ at 200-mW laser power for 10 s with 300 mm/s of maximum scanning speed^14^. However, it is insufficient for application to an R2R process, even with a complex mirror system for a large scale, due to its small sintering spot size.

An IPL technique was highlighted in recent research with excellent results in terms of conductivity improvement with a short sintering time, absence of substrate damage, R2R compatibility, and large sintering area^15^,^16^,^17^,^18^. However, the performance achieved by Hösel *et al*.^15^ was apparently insufficient for a resistivity of 6.83 Ω/□ with a voltage of 2.8 kV and web speed of 1 m/min compared with that of the other sintering methods. The technique was implemented on an inline R2R process for an Ag material with high transportation speed. Other IPL cases showed extremely fast and effective sintering results within milliseconds using copper (Cu) NP^16^, Cu oxide NP^17^, and Cu nanowire^18^. Despite these advantages, IPL is the most expensive technique to install in the R2R process. Furthermore, overheating and substrate damage can occur unless the IPL-sintering conditions are properly tuned for the employed inks. Moreover, the tuning of IPL-sintering conditions is complicated due to the narrow operating window in a millisecond-scale sintering duration. On the other hand, microwaves, which are commonly used in daily life, can be used to achieve high-speed sintering and partially set an inline process. Perelaer *et al*., for example, reported a sheet resistance of 2 Ω/□ based on experimental conditions with a frequency of 2.45-GHz and power of 1 W for 1 s^19^. Nonetheless, the technique cannot cover a large area.

Among the above-mentioned sintering methods, IR is the most suitable owing to its relatively lower cost, R2R compatibility, easy accessibility, and parameter optimisation of the inline process. Therefore, an IR-sintering module was applied in the present study to R2R gravure printing systems for large-area, high-speed, and substrate-damage-free sintering. Excellent thermal-sintering performance of 11–35% of bulk Ag conductivity with gravure-printed Ag patterns was obtained from our previous study^9^. A gravure printing technique commonly used in R2R systems was selected for Ag deposition owing to its high printing speed and patterning ability on a large scale^6^,^9^,^20^.

Two types of pastes—Ag NP and Ag flake—have been employed in R2R gravure printing^9^,^21^,^22^,^23^ in which a tailing and spreading defect can occur during the lengthy printing process^9^, which explains why the doctoring of a flake paste is easier than that of NP in terms of the size differences. If the flake can obtain compatible electrical performance as that of the NP case, it could be used as a good process example. Furthermore, Hrehorova *et al*. reported that the Ag flake result is superior to that of the Ag NP owing to its large pattern thickness^23^. The use of Ag NPs and Ag flake is necessary for the sintering process. Accordingly, the sintering results of the Ag NP and Ag flake were compared. The large-area-printed Ag patterns were sintered under IR conditions of sintering time and determined by the web velocity, distance between the substrate and IR lamp, and IR lamp power. On the basis of the statistical analysis of the IR sintering results, the optimised process parameters were analysed and proposed using analysis of variance (ANOVA).

## Results and Discussion

### Gravure printing

**Figure 1:**
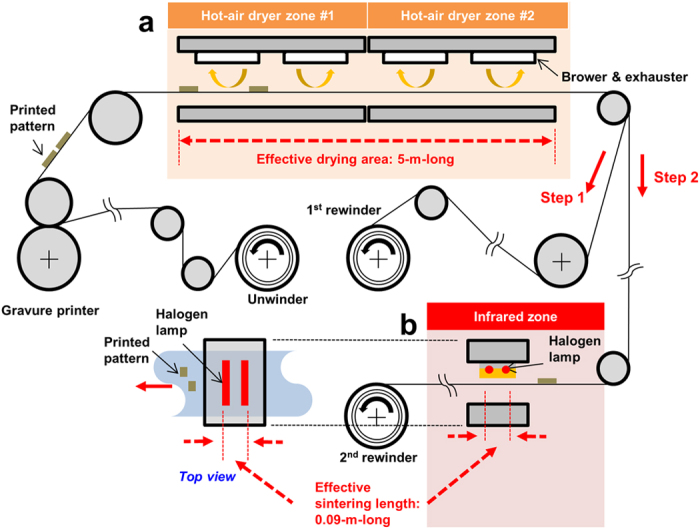
R2R gravure printing of the Ag square pattern (step 1) and sintering process (step 2) using (**a**) hot air and (**b**) IR module.

**Figure 2:**
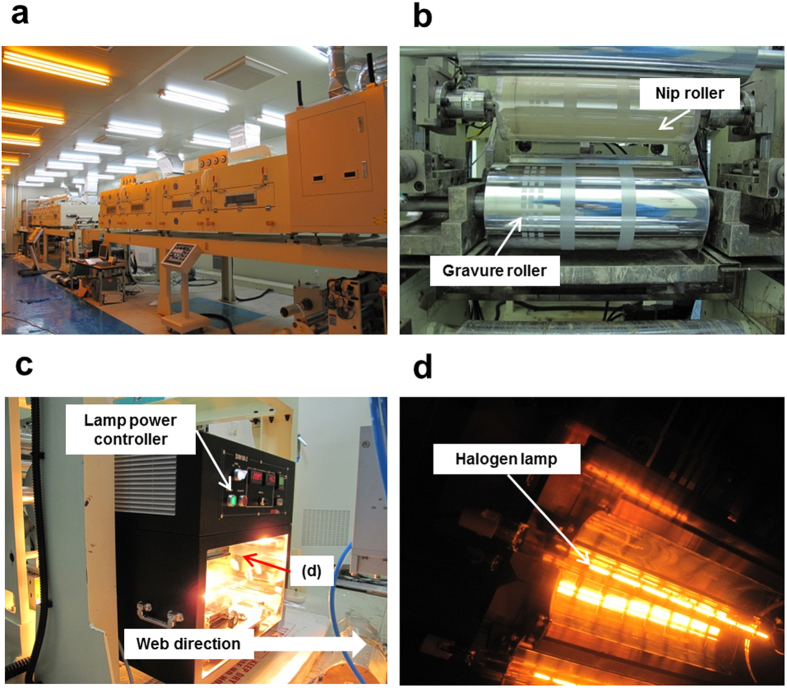
(**a**) Two-layer gravure printing system at Konkuk University, Seoul, Korea. (**b**) Gravure section with nip roller and cylinder for square pattern. (**c**) Inline IR module designed for web transportation. (**d**) IR lamp upper Ag-printed PI substrate.

The printing and sintering processes were separately performed to independently control the sintering conditions using identical printed patterns. Normally, the sintering process is directly conducted after the printing process for simultaneous drying and sintering, as performed in previous research^24^. However, the sintering process in this work was independently performed from the drying process after the gravure printing process to observe only the sintering effect among the samples. Two rewinding processes were involved, namely, printing (step 1) and sintering (step 2), as shown in Fig. 1. The Ag NP and Ag flake pastes were printed and dried on a polyimide (PI) film using a direct gravure printer [Fig. 2(b)] at 75–80 °C for 1 min of drying time (5 m/min velocity through a 5-m-long drying section), as shown in Fig. 1(a). The Ag NP and Ag flake had average sheet resistances of 45.2 and 160.6 Ω/□, respectively. The initially printed pattern was dried to obtain the samples to confirm the effectiveness of the IR sintering. The minimum treatment did not involve staining using idle rolls.

### IR sintering

The sintering process (step 2) was conducted after the printing process under various process conditions [Fig. 1(b)]. The IR lamp power, distance between the IR lamp and substrate, and sintering time could be easily controlled in the inline process. The experimental parameters and conditions are listed in Table 1. The main parameters were screened by referring to previous research^24^; however, specific ranges of the sintering parameters needed to be optimised by considering the target devices^25^. Control of the sintering time was easy by determining the web velocity in the human–machine interface (HMI) of the R2R system. The web velocity is the operating velocity of the substrate, which is the same as that of the tangential velocity of the rollers. Accordingly, the web velocity was set to five levels—0.5, 1, 2, 3, and 5 m/min—at 10.8, 5.4, 2.7, 1.8, and 1.08 s sintering times, respectively, by considering a 0.09-m-long effective IR lamp module. Moreover, the initially printed layer was also sintered in a hot-air dryer at 100 °C for 15, 30, and 60 s with 10, 5, and 2.5 m/min web velocities (effective drying length of 5 m), respectively, for comparison with the IR cases. (For the full factorial experimental table of the IR and hot-air experimental table, please refer to Tables S1 and S2 in the Supplementary Information.) On the basis of the full factorial table in Table S1, the experiment was carried out using 90 sets of Ag NPs and Ag flakes.

### Ag NP layer

**Figure 3:**
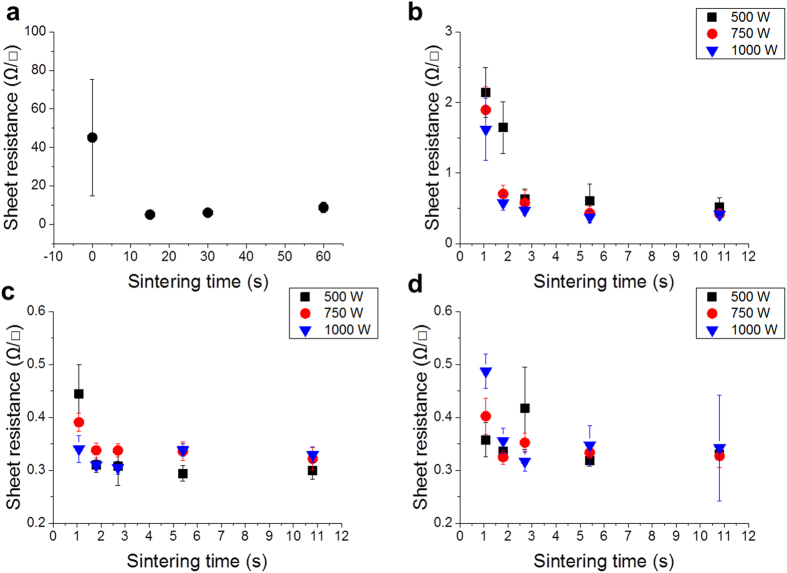
Sheet resistance of the Ag NP layer: (**a**) hot-air sintering at 100 °C and IR sintering with various powers at distances of (**b**) 70 mm, (**c**) 50 mm, and (**d**) 20 mm.

**Figure 4:**
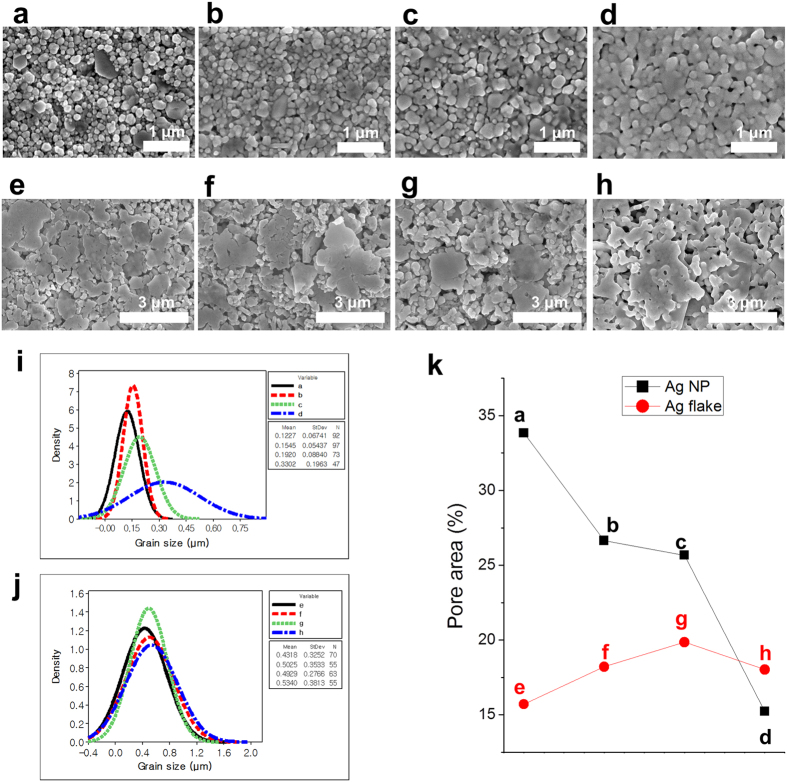
SEM images of the printed and sintered layers of the (**a**–**d**) Ag NP and (**e**–**h**)Ag flake. (**a**) Hot air using Ag NP sample at 100 °C temperature and 60-s sintering time and IR-sintered layer with the following distances, IR lamp powers, and sintering times: (**b**) 50 mm, 500 W, and 1.08 s, respectively; (**c**) 50 mm, 500 W, and 5.4 s, respectively; and (**d**) 20 mm, 1000 W, and 1.08 s. respectively. (**e**) Hot-air-dried Ag flake layer at 100 °C temperature and 60-s sintering time and IR-sintered layer with distances, IR lamp powers, and sintering times of (**f**) 70 mm, 500 W, and 1.08 s, respectively; (**g**) 20 mm, 500 W, and 1.08 s, respectively; and (h) 20 mm, 1,000 W, and 10.8 s. Particle size calculation and fitted distribution of this histogram for (**i**) Ag NP and (**j**) Ag flake. (**k**) Pore area calculation based on image processing and graph of the pore area percentage as increments in the sintering conditions.

The experimental results of the Ag NP are shown in Fig. 3. The initial sheet resistance was obtained with a high standard deviation of 45.15 ± 30.23 Ω/□. For the hot-air drying system, the temperature was increased up to 100 °C for 15, 30, and 60 s. The corresponding sheet resistances decreased to 5–8.75 Ω/□, as shown in Fig. 3(a). The scanning electron microscope (SEM) image of the hot-air-dried Ag layer shows the distinct individual Ag NP in Fig. 4(a). The results of the IR sintering cases are shown in Fig. 3(b–d) as a function of the distance between the IR lamp and substrate. The sheet resistance markedly decreased from 45.15 to 2.14 Ω/□ when the conditions were as follows: sintering time of 1.08 s, power of 500 W, and distance of 70 mm, as shown in Fig. 3(b). The farthermost distance of 70 mm shows a general decreasing tendency of the sheet resistance when the lamp power and sintering time are increased. In contrast, the sheet resistances at 50- and 20-mm distances further decrease to 0.445 and 0.357 Ω/□, respectively, at 1.08 s of sintering time and 500 W of lamp power, as shown in Fig. 3(c,d). Moreover, the lowest average sheet resistance obtained is 0.294 Ω/□. The sintering time, lamp power, and distance are 5.4 s, 500 W, and 50 mm, respectively, as shown in Fig. 3(c).

Figure 3(c) shows that the 500-W power condition can yield a lower sheet resistance. Furthermore, large instability exists in the 20-mm-distance result, as shown in Fig. 3(d). The 1,000-W IR lamp power and long sintering duration indicate a large standard deviation of approximately 0.2 Ω/□. These results show that the severe sintering power of the IR lamp with a short distance to the substrate leads to instability in the printed pattern owing to delamination (see Figure S1 in the Supplementary Information for delamination of the Ag NP surface). In particular, the 20-mm distance is too close to the printed pattern; thus, the sheet resistance variation is very high and unpredictable even if the process conditions are controlled. In addition, the best result of fast sintering yields a sheet resistance of 0.34 Ω/□ at a 50-mm distance and IR lamp power of 1,000 W.

### Ag flake layer

**Figure 5:**
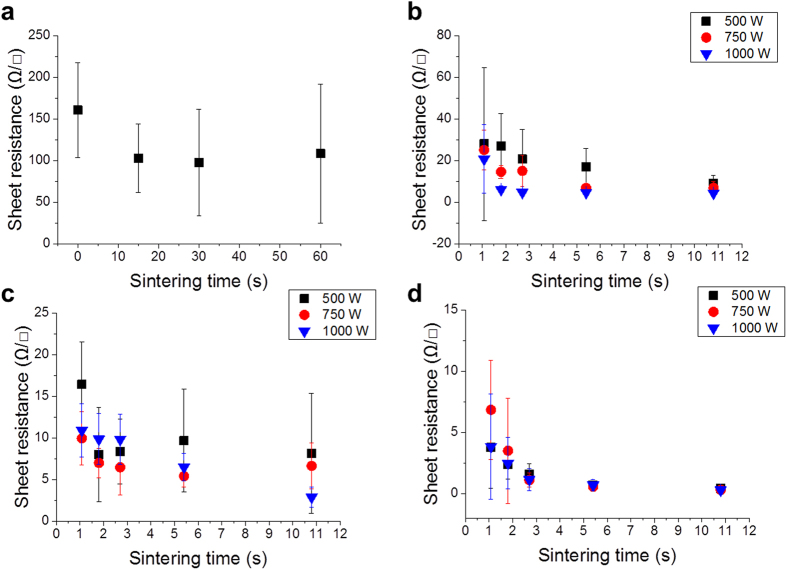
Sheet resistances of the Ag flake layer by (**a**) hot-air sintering at 100 °C and IR sintering as a function of the distance at (**b**) 70 mm, (**c**) 50 mm, and (**d**) 20 mm.

All results of the Ag flake layers generally exhibit high deviation in the sheet resistance, as shown in Fig. 5. The Ag flake layer was not easy to sinter. It required a long duration of IR sintering and high exposure energy of the IR lamp, in contrast to the Ag NP layer, because of the large particle size (small surface area) of 1–10 μm compared with that of the Ag NP^9^. The initial sheet resistance of the Ag flake layer is 160.5 ± 57.3 Ω/□. As the hot-air sintering temperature increases, a high deviation and a small reduction in the sheet resistance occur, as shown in Fig. 5(a). The SEM image of the Ag flake sample [Fig. 4(e)] for hot-air sintering shows a mixed size of Ag flakes, as shown in Fig. 4(j). However, in the case of flake aggregation between the large- and small-sized particles from 431 to 534 nm (mean value) with a 330-nm standard deviation, a low sheet resistance is obtained, as shown in Fig. 4(h). IR sintering is also very effective for Ag flake layers, as shown in Fig. 5(d).

Figure 5(a–d) show that the maximum IR lamp power of 1,000 W indicates a low standard deviation compared with the 500 and 750 W. Additionally, the decreasing ratio of 1,000-W power is steeper than that of the IR lamp power. Figure 5(c) shows that the 50-mm-distance condition exhibits the particular propensity of the sheet resistance with increasing sintering time using different lamp powers. However, a minimal increase and a high standard deviation in the sheet resistance appear at 500 and 750 W. The shortest distance of 20 mm exhibits a stable decrement in the sheet resistance; hence, 3.838 and 0.288 Ω/□ are obtained under the condition of maximum voltages with sintering times of 1.08 and 10.8 s, respectively, as shown in Fig. 5(d). The SEM images of the best results are shown in Fig. 4(g,h).

For in-depth evaluation, image processing was conducted to calculate the pores among the particles (see Figure S2 in the Supplementary Information for the SEM image pore calculation based on image processing and the results). The quantified result of the Ag NP is indicated by the decreasing pore tendency with a densely growing particle area by agglomeration, as shown in Fig. 4(k). On the other hand, the flakes do not show significant differences in their SEM images. Evidently, the Ag NP has a greater effect than the Ag flake under the same IR energy intensity.

The effect of sintering on the Ag NP is shown in the narrow box area with 75% data in Figure S3. However, the flake case is also indicated by a narrow shape on the thickness in the box plot after sintering. All thickness values become thicker than those in the cases before sintering. By considering the immense deviation in the cases with flakes, the sintering effect on the pattern thickness is difficult to confirm due to its nominal deviations.

Figure 4(i,j) also show that the Ag NP presents clear shifting of the grain size to a high value according to the IR energy. In contrast, the fitted distribution line of the flakes does not indicate immense large difference. Instead, it shows that the mean value changes from 431 to 534 nm in the shifting of the grain size. The difficulty of clear aggregation in the flake case is presumed to be due to the large thickness of the samples and the high-energy requirement for a low surface area.

### Transmittance spectra

The IR lamp employed in this study has a relatively strong intensity in the wavelength range from 800 to 2000 nm. The light transmittance of the printed layer can affect the performance of the IR sintering. In the work of Cherrington^24^, a comparison of the transmittance between the wet and dry states of the Ag layer was conducted. In the present study, the initially dried pattern was compared with the IR sintered pattern under different distances. The other sintering conditions were fixed at 1,000 W of IR lamp power and 10.8 s of sintering time.

**Figure 6:**
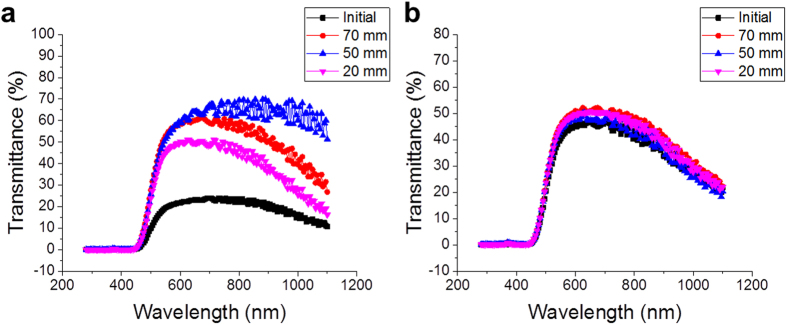
Transmittance spectra of the IR-sintered layer of (**a**) Ag NP layer and (**b**) Ag flake layer as a function of the distance at 20, 50, and 70 mm with 1,000-W IR lamp power and 10.8-s duration.

**Figure 7:**
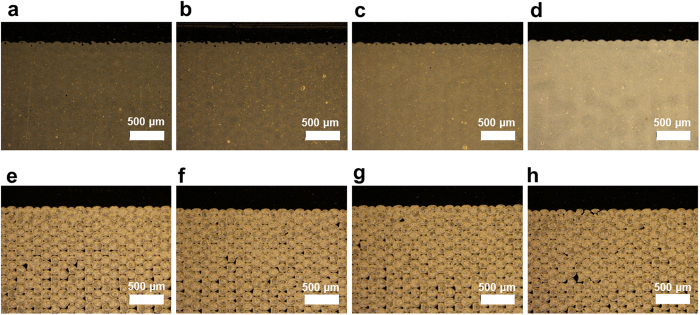
Microscope images of the gravure printed layer of (**a**–**d**) Ag NP and (**e**–**h**) Ag flake. (**a,e**) Initially dried Ag layers were sintered under the following conditions: distance, IR lamp power, and sintering time of (**b**,**f**) 70 mm, 1,000 W, and 10.8 s, respectively; (**c,g**) 50 mm, 1,000 W, and 10.8 s, respectively; and (**d,f**) 20 mm, 1,000 W, and 10.8 s respectively.

Figure 6(a) shows a comparison of the transmittance spectra of the Ag NP layer. The initially dried pattern is distributed in the range of approximately 20% (peak). After IR radiation at a 70-mm distance, it is increased to 60% transmittance. The lower transmittance of the printed layer indicates high absorption of energy^12^. The distance of 50 mm increases the transmittance up to 70%. However, the transmittance decreases at a distance of 20 mm. This transmittance decrement may be the result of increased reflection of the sintered metallic film^26^. This phenomenon can be confirmed in the microscope image with a measurement setting under the same light intensity shown in Fig. 7(a–d). The Ag NP is shown by the dark colour at the initial pattern, as shown in Fig. 7(a). As the distance decreases from 70 to 20 mm, the colour of the Ag layer changes to a bright appearance. It is presumed to be the most sintered layer close to a pure metal film; thus, reflectivity increases (see Table S3 and Figure S4 in the Supplementary Information for the analysis of all Ag layer results in the transmittance under different conditions).

In contrast, the Ag flake pattern shows similar transmittance in every distance, as shown in Fig. 6(b), which means that the IR energy is consistently absorbed in every sample. The microscope image of the flake layer shows a similar colour with the change in the sintering conditions. We consider that the large-sized particles of the Ag flake paste are pinned; thus, aggregating them is not as easy as that in the previous SEM images. Moreover, the patterning quality of the Ag flake layer is worse than that of the Ag NP layer due to the pinholes, as shown in Fig. 7(e–h).

The surface topography was investigated to identify the transmittance changes with bright appearances on the Ag NP layer. The interferometer measured the sintered thickness of the Ag NP and Ag flake as 2.23 ± 1.19 and 6.02 ± 2.07 μm, respectively. Moreover, the roughness of each case is indicated as 209 ± 52 nm and 3.08 ± 0.64 μm, respectively. No sample indicates a meaningful tendency in the roughness value with the energy density. (Please see Figure S5 in the Supplementary Information for the interferometer-measured 3-D profile of the gravure printed and sintered patterns as well as its contour plot of the roughness value as a function of the calculated relative energy density and thickness.) Thus, we presume that the colour change in the microscope image and transmittance value could be due to the change in the material properties in the binder or Ag materials and not to the roughness or thickness changes in the sintered patterns.

### ANOVA

A statistical approach was introduced to analyse the performance of the IR sintering process. The ANOVA of the sheet resistance versus distance (A), power (B), and sintering time (C) for the Ag NP and Ag flake layers was performed. The set of parameters is listed in Table 1 and Table S1. Full factorial design, the most common and effective technique to confirm the significance of experimental conditions, was implemented^25^,^27^,^28^. Table 2 lists the degree of freedom (DF), sequential sums of squares (Seq SS), adjusted SS (Adj SS), adjusted mean squares (Adj MS), F-value, standard error (S), and R-square value of the Ag NP and Ag flake layers. Here, the F-values indicate the significance and criticality of the distance (A), power (B), and sintering time (C). The interaction effect of ABC was very small; hence, it was pooled in this analysis. All results were derived by the MINITAB commercial software based on the experimental results.

The F-values of the Ag NP layer show that the distance (A) and sintering time (C) are the most significant factors for the main effects. The interaction between the distance and sintering time (AC) is critical to the sheet resistance. The interaction between the distance and IR lamp power (AB) is higher than the main effect of the IR lamp power (B) factor. The interaction between the IR lamp power and sintering time (BC) exhibits a low interaction effect, which implies that the Ag NP is hardly sintered. Moreover, the sintering time increases with high exposure energy regardless of the distance (A). As a result, the performance of the Ag NP layer sintering influences the distance and sintering time more than the IR lamp power. On the other hand, the Ag flake layer shows a large effect on the distance (A), similar to the NP case; however, it presents a similar main effect on the IR lamp power (B) and sintering time (C). The difference in the case of the Ag NP is the interaction of each factor. The interaction between distance (A) and IR lamp power (B) is higher than that between distance (A) and sintering time (C) in the Ag flake layer. The IR lamp power is maximised when the distance decreases. The interaction between IR lamp power (B) and sintering time (C) is also low in the Ag flake layer.

In summary, the Ag NP and Ag flake show similar tendencies in terms of the main effect. However, the effects of the interactions do not agree with each other. Similar to the previous research on the laser-sintering process, longer laser treatment under the same calculated energy density is superior to shorter laser time exposure with a higher laser power^17^. In the flake case, as mentioned earlier, the surface-volume ratio is low; thus, steady energy application is more effective on the Ag flake-printed pattern. Whereas the Ag flake layer is sintered in proportion to the main effects, the Ag NP layer is already entirely sintered at the low IR lamp power and middle distance condition owing to its high surface-volume ratio. Therefore, excessive increase in the IR lamp power does not contribute to the sheet resistance of the Ag NP layer. Instead, it generates over-sintering, reflection, and convergence to the specific value. (Please see Figure S6 in the Supplementary Information for the main effect and contour plots of all samples resulting in the sheet resistance under different conditions).

### Energy density

**Figure 8:**
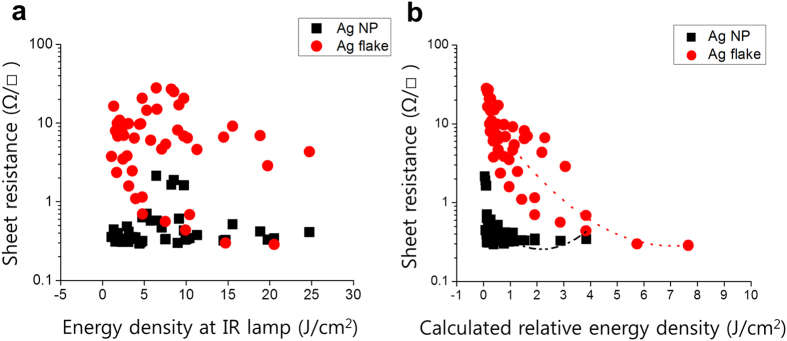
Average sheet resistance values of all experimental cases as (**a**) energy density at the IR lamp and (**b**) calculated relative energy density (with polynomial curve fitting).

The sheet resistance values are shown in Fig. 8 with the calculated relative energy density. The energy density at the IR lamp was derived by Sowade *et al*. as Equation (1) 12, where *E*_*D*_, *P*_*D*_, and *D*_*e*_ are the energy exposure density, power density, and duration of exposure, respectively.
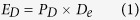


This equation considers the energy exposure density from the emitter. Thus, estimating the energy density on the substrate is not possible without measuring the actual energy at the surface. As mentioned earlier, the distance between the substrate and IR lamp is the most significant factor for determining the sheet resistance. For the mapping of the energy density on the substrate, a weight factor is applied to Equation (1) to express the simple relative energy density as Equation (2). This equation can be used to express the energy density at the substrate, where *F*_*d*_, *F*_*P*_, *F*_*t*_, and *α* are the F-value ratio of distance (A) and power (B), sintering time (C), and weighting factor of the distance to meet the physical meaning (*α* *=* *1/d*, where *d* is the perpendicular distance between the substrate and IR lamp), respectively. The interaction effect can be omitted because the main effect is sufficiently large.
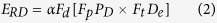


Figure 8 shows the plots of the sheet resistance values under all experimental conditions using Equations (1) and (2). We confirm that the sheet resistance of the Ag NP is not affected by the energy densities from the IR lamp, as shown in Fig. 8(a). However, as mentioned earlier, we cannot reasonably explain the effect of the lamp energy density on the Ag patterns. By considering the relative energy density, the sheet resistance of the Ag NP is inversely proportional to the relative energy density compared with that of the Ag flake pattern, as shown in Fig. 8(b).

The sheet resistance of the Ag NP pattern rapidly decreases as the relative energy density increases. The increased energy does not significantly affect the Ag NP pattern. At low energy, the Ag NP pattern has a high deviation distribution; however, it decreases with small increments in the energy density. Interestingly, the Ag flake pattern constantly decreases as the relative energy density of 7.66 J/cm^2^ increases, and it finally achieves the lowest sheet resistance value of 0.288 Ω/□ at 1,000-W power, 20-mm distance, and 10.8-s sintering time. In contrast, the Ag NP cases indicate similar sheet resistance values under different relative energy densities in the range of 0.06–3.85 J/cm^2^.

In summary, most of the lowest sheet resistance values are distributed in the range of strong sintering conditions; however, they are most effective in the flake cases and not in the NP cases. These results can be correlated with the description in the previous section on the analysis of over-sintering with a high deviation in the sheet resistance and reflection.

In terms of absolute electrical performance, the results of this research can be summarised as follows: The sheet resistance is significantly improved from the initial value to the final value; however, the thickness values are not sufficient when resistivity is considered. In gravure printing, the printed pattern is relatively thicker than those in the other studies. Most laboratory-scale printed patterns using the inkjet method in previous studies have a 50–400-nm thickness^11^,^12^,^13^,^14^,^16^. Thus, the effect of sintering can be stronger on a thin layer than on a thick layer. Moreover, the bumpy surface of the gravure printed layer is caused by the dot-type patterning on the gravure cylinder, which aggravates the electron movements and increases the electron traps^9^. It thus causes poor resistivity on the gravure printed pattern. Nevertheless, under a longer sintering condition of 10.8 s, the flake obtains the best result of 0.288 Ω/□. This value is a 147222% improvement from the initial sheet resistance value of 424 Ω/□. These results surpass those of the previous studies on R2R-compatible processes, such as IR^12^, IPL^15^, thermal^9^, and UV^13^ approaches in terms of sintering time, performance, and high throughput (please see the Table S4 comparison of performance with other studies). It is significant that the experiment was continuously performed on a large-scale R2R system because the required resistance is important for application development to decrease both the sintering temperature and resistivity.

## Conclusions

In conclusion, fast and effective IR sintering has been performed in a large-area R2R gravure printing process. Gravure printing and sintering processes were conducted for a long duration using numerous samples. These samples enabled statistical analysis. Therefore, owing to the correlations among the process conditions—IR lamp power, distance from the IR lamp to printed patterns, and exposure time—the sheet resistances of the two types of pastes, namely, Ag NP and Ag flake, could be identified. From the experimental data, the calculated energy density was proposed by considering not only the lamp power and sintering time but also the distance term in the sintering process. Different tendencies of and effects on the Ag NP and Ag flake were identified in terms of the transmittance and particle sizes. The Ag NP printed pattern is effective for fast reduction of sheet resistance due to the nanometer-sized particles and high absorbance rate in the nominal printed pattern. However, a considerable energy density destabilises the Ag NP pattern. This phenomenon is confirmed by the high standard deviation of the sheet resistance value and unexpected tendency among the experimental conditions. On the other hand, the flake printed pattern shows a steady improvement in the sheet resistance as the energy density increases. Unlike the Ag NP, the Ag flake has large-sized particles, which produce consistent transmittance. Thus, the sheet resistance decreases as the energy density increases. As a result, the distance between the substrate and IR lamp is the most effective parameter in every sample, whereas the second most effective one is the sintering time. The lamp power is not significant in the Ag NP; however, it is significant in the Ag flake layer. These guidelines and issues of process conditions can be applied to industrial areas. Furthermore, the sheet resistance can be improved by upgrading the module in the R2R process.

## Experimental Section

### Materials and Method

The Ag NP paste was purchased from Paru Co., Ltd. The model name, particle type, particle size, metal contents, viscosity, main solvent, and boiling point of the solvent were PG-007, NP, 20–100 nm, 60%, 15000 cP, dipropylene glycol, and 230.5 °C, respectively. The Ag flake paste model was FTL-770; it was purchased from Fine Paste Co. Ltd. The specifications of FTL-770 are as follows: particle type, size, viscosity, solvent, and boiling point of the solvent of flake, 1–10 μm, 20,000 cP, diethylene glycol dibutyl ether, and 256 °C, respectively. The substrate was a PI film purchased from Kolon Co. Ltd. It was 300-mm wide and 25-μm thick and had a 321-nm surface roughness and a coefficient of thermal expansion of 25.51–30.27 μm/(m·°C) based on the ASTM E831 test.

In the gravure printing process, the operating velocity, operating tension, nip, and doctor pressure were set to 5 m/min, 1 kgf, 0.5 MPa, and 0.6 MPa, respectively. The printed pattern was square-shaped with a dimension of 7.5 × 7.5 mm^2^. After the printing process, hot-air drying was performed at 75–80 °C for 1 min. An additional sintering process using a hot-air module was performed at 100 °C for 10–60 s with 2.5–15 m/min web velocity for comparison. IR sintering was conducted under the following conditions: 0.5–5 m/min web velocity, 10.8–1.08 s sintering time, IR lamp powers of 500, 750, and 1,000 W, and distances between the IR lamp and substrate of 20, 50, and 70 mm.

### R2R system

The R2R gravure printing system (Sung An Machinery Co., Ltd.) was composed of an unwinder, infeeder, preheater, direct gravure printer, hot-air dryer (convection heat transfer, 150 °C maximum temperature, and effect sintering area of 300 × 5000 mm^2^), lateral guider, outfeeder, cooler, and rewinder unit. The R2R system had a maximum range of operating tension of 20 kgf and an operating velocity of 50 m/min. The web substrate was controlled by several load cells and a lateral web guider (Fife Co., Ltd.).

The IR sintering module was manufactured by DTX Co., Ltd. The module consisted of two IR lamps. The supply voltage, maximum IR lamp power, output wavelength, lamp diameter, lamp length, and effective sintering area were 207.9 V, 500 W, 0.8–2 μm, 14 mm, 200 mm, and 200 × 90 mm^2^, respectively. The IR module power could be controlled by the voltage setting. The voltages were set to 148, 174.7, and 207 V with 3.37, 4.29, and 4.83 A for the 500, 750, and 1000 W of IR lamp power, respectively.

### Characterisation

The thickness and roughness of the printed pattern (root mean square value) were measured using an interferometer (NV-2000, Nanosystems Co., Ltd.) The sheet resistance was measured using a source meter (2611 A, Keithley Co., Ltd.). A Cu tape was attached to the side of the printed Ag square pattern. Ag NP was deposited to reduce the contact resistance between the Ag layer and Cu tape. Then, the sheet resistances were measured using two probes. The sheet resistances were measured 20 times for each sample; then, the average and standard deviation were calculated for analysis. The surface morphology was measured by a microscope (LV100ND, Nikon Co., Ltd.) with a constant intensity of the layer for comparison of each pattern. The SEM images were measured by field emission SEM equipment (S-4800, Hitachi, Co., Ltd.) with magnification power of 30,000 (Ag NP) and 15,000 (Ag flake) times. The grain and gap size were measured using the linear intercept method based on Matlab software^29^. Moreover, the pore area was calculated using the ImageJ open-source program based on the particle size analysis and colour threshold. The transmittance of the printed layer was measured using a UV/Vis spectrophotometer (2120 UV, Optizen Co., Ltd.) with a wavelength range of 280–1100 nm. For the statistical analysis, the full factorial methodology was conducted by MINITAB commercial software. The distance (A), IR lamp power (B), and exposure time (C) were selected for the ANOVA test of the sheet resistance of the Ag layer. The interaction effect of all experimental parameters (ABC) was not significant; thus, it was pooled in the ANOVA test.

## Additional Information

**How to cite this article**: Park, J. *et al*. Fast sintering of silver nanoparticle and flake layers by infrared module assistance in large area roll-to-roll gravure printing system. *Sci. Rep.* **6**, 34470; doi: 10.1038/srep34470 (2016).

## Supplementary Material

Supplementary Information

## Figures and Tables

**Table 1:** Screening of the main experimental parameters.

Parameter (mark)	Unit	Set value (level)
Distance between substrate and IR lamp (A)	mm	20 (−1), 50 (0), 70 (+1)
IR lamp power (B)	W	500 (−1), 750 (0), 1000 (+1)
Sintering time (C)	s	1.08 (−2), 1.8 (−1), 2.7 (0), 5.4 (+1), 10.8 (+2)

**Table 2:** ANOVA of the sheet resistance, distance, IR power, and sintering time (exposure time) of Ag NP and Ag flake layers.

Paste	Source	DF	Seq SS	Adj SS	Adj MS	F
Ag NP	A	2	2.74782	2.74782	1.37391	75.76
	B	2	0.14697	0.14697	0.07349	4.05
	C	4	1.79056	1.79056	0.44764	24.69
	AB	4	0.32149	0.32149	0.08037	4.43
	AC	8	2.65576	2.65576	0.33197	18.31
	BC	8	0.13074	0.13074	0.01634	0.90
	Error	16	0.29014	0.29014	0.01813	
	Total	44	8.08348			
	S = 0.134663; R^2^ = 96.41%					
Ag flake	A	2	1097.336	1097.336	548.668	70.03
	B	2	179.637	179.637	89.818	11.46
	C	4	491.187	491.187	122.797	15.67
	AB	4	226.980	226.980	56.745	7.24
	AC	8	185.283	185.283	23.160	2.96
	BC	8	16.159	16.159	2.020	0.26
	Error	16	125.349	125.349	7.834	
	Total	44	2321.932			
	S = 2.79899; R^2^ = 94.60%					
