# Supplementary material for: Fast sintering of silver nanoparticle and flake layers by infrared module assistance in large area roll-to-roll gravure printing system
Source: Sci Rep. 2016 Oct 7;6:34470. doi: 10.1038/srep34470 (PMC5054385; doi:10.1038/srep34470)
Supplement: Supplementary Information [file srep34470-s1.doc]

**SUPPLEMENTARY INFORMATION**

**Fast sintering of silver nanoparticle and flake layers by infrared module assistance in large area roll-to-roll gravure printing system**

Janghoon Park^1^, Hyi Jae Kang^1^, Kee-Hyun Shin^1,2^ & Hyunkyoo Kang*^,3^

^1^Department of Mechanical Design and Production Engineering, Konkuk University, Seoul, Korea

^2^Flexible Display Roll-to-roll Research Center, Konkuk University, Seoul, Korea

^3^Digital Printing and Imaging Technology, Technische Universität Chemnitz, Chemnitz, Germany.

*Corresponding author: hyunkyoo@gmail.com


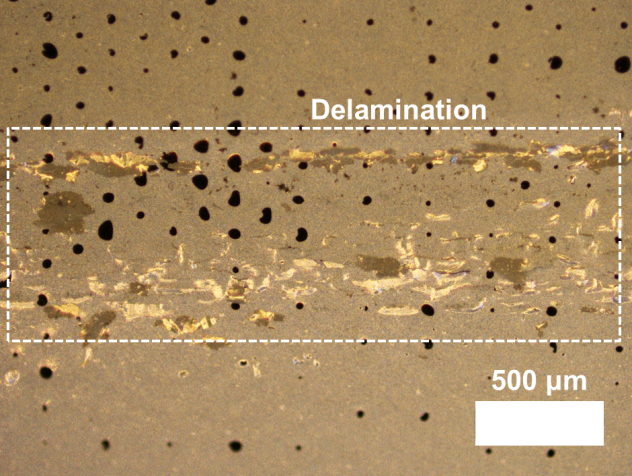


**Figure S1.** Delamination generation on the Ag NP surface.

Figure S1 shows the delamination on the Ag NP-printed and IR-sintered surface. The microscopic photograph indicates the sintering conditions of 20 mm, 1,000 W, and 1.08 s of distance, power, and exposure time, respectively. This delamination naturally occurs on the surface, which implies that the adhesion between the Ag layer and substrate worsens when the samples are exposed to high energy.


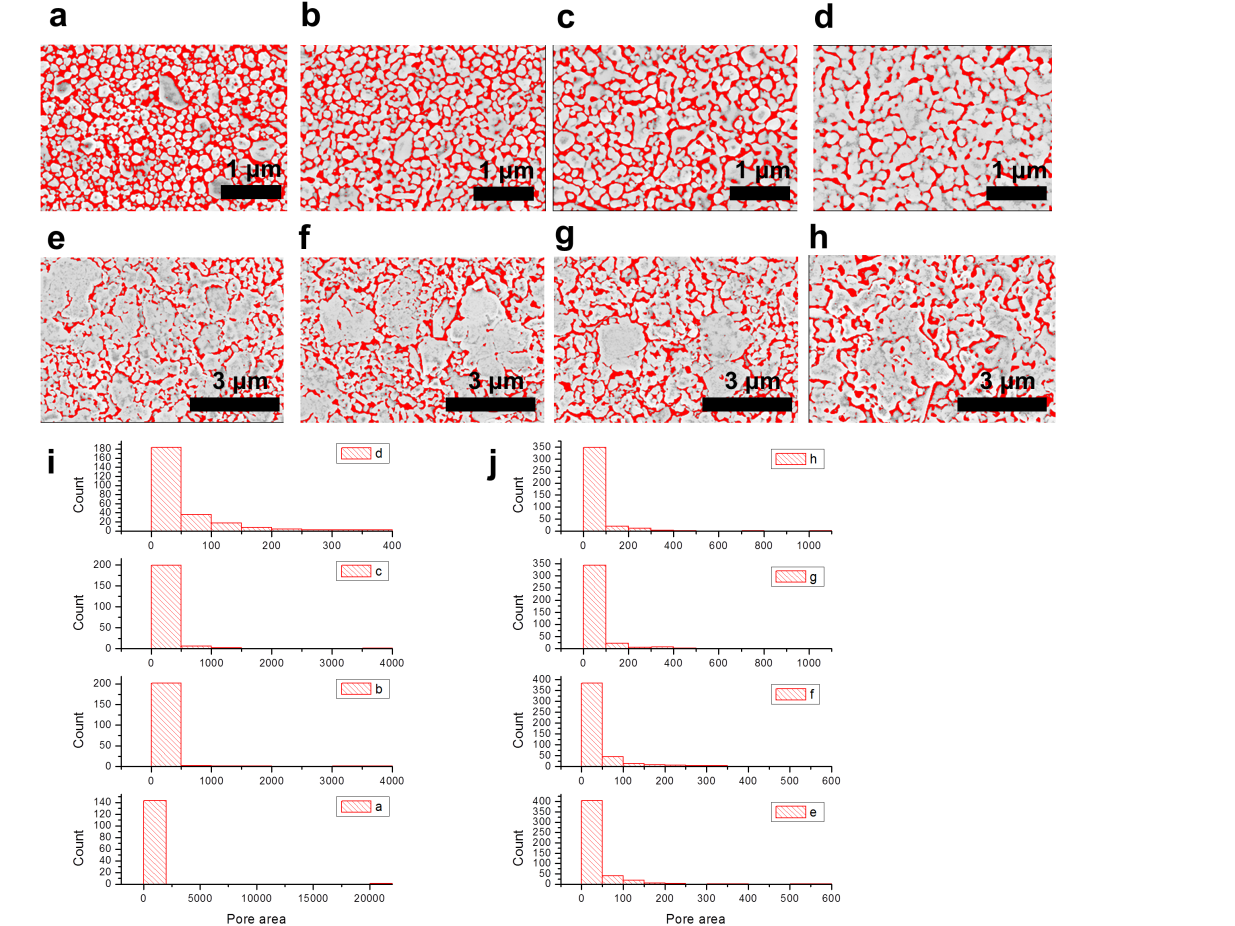


**Figure S2**. Pore calculation of the SEM image based on image processing for the printed and sintered layers of (a)–(d) Ag NP and (e)–(h) Ag flake. Pore area calculation based on the red colour area of the (i) Ag NP and (j) Ag flake results.

Figure S2 shows the pore calculation of the SEM images shown in Figure 4 of (a)–(d) Ag NP and (e)–(h) Ag flake. Based on the image processing, the pores between the particles are presented in red. According to the red area, all areas shown in Figures S2 (i) and (j) are histograms. These results are shown as a pore-area plot in Figure 4(i).


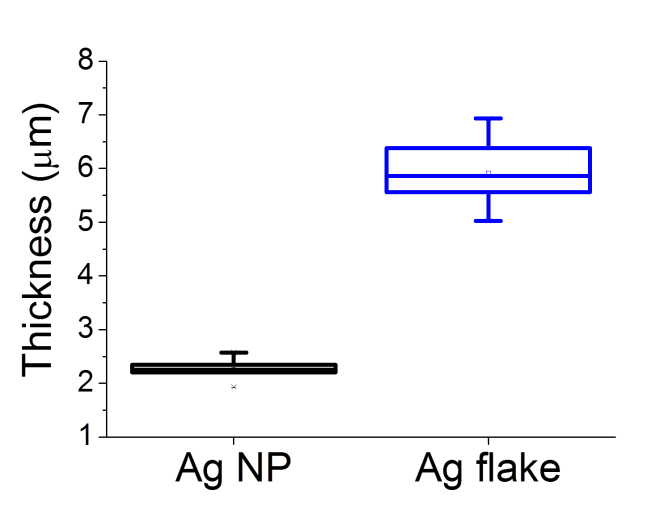


**Figure S3**. Thicknesses of the Ag NP and Ag flake-printed patterns before and after sintering.

Figure S3 shows that before and after sintering, Ag NP thicknesses of 2.25 ± 0.17 μm and 2.23 ± 1.19 μm, respectively, are obtained. In contrast, the Ag flake layers show thicknesses of 5.03 ± 0.83 μm and 6.02 ± 2.07 μm in the absence and presence of the sintering process, respectively. In the gravure process, a thicker layer is obtained for comparison with the employed inkjet process layer. Thus, the sintering effect on the thickness of the layer is difficult to confirm due to its nominal deviations.

Table S1 lists the experimental setup for the full factorial analysis. It shows that the experiment was carried out 45 times using two types of Ag NP and Ag flake. Thus, the total number of experiments is 90. Table S2 lists the comparison performed in the existing hot-air dryer.

**Table S1** Full factorial experimental table of Ag NP and Ag flake

| **Experiment order** | **Experiment level (ABC)** | **Distance (mm)** | **Lamp power (W)** | **Exposure time (s)** | **Velocity (m/min)** |
| --- | --- | --- | --- | --- | --- |
| 1 | -1 -1 +2 | 20 | 500 | 10.8 | 0.5 |
| 2 | -1 -1 +1 | 20 | 500 | 5.4 | 1 |
| 3 | -1 -1 0 | 20 | 500 | 2.7 | 2 |
| 4 | -1 -1 -1 | 20 | 500 | 1.8 | 3 |
| 5 | -1 -1 -2 | 20 | 500 | 1.08 | 5 |
| 6 | -1 0 +2 | 20 | 750 | 10.8 | 0.5 |
| 7 | -1 0 +1 | 20 | 750 | 5.4 | 1 |
| 8 | -1 0 0 | 20 | 750 | 2.7 | 2 |
| 9 | -1 0 -1 | 20 | 750 | 1.8 | 3 |
| 10 | -1 0 -2 | 20 | 750 | 1.08 | 5 |
| 11 | -1 +1 +2 | 20 | 1000 | 10.8 | 0.5 |
| 12 | -1 +1 +1 | 20 | 1000 | 5.4 | 1 |
| 13 | -1 +1 0 | 20 | 1000 | 2.7 | 2 |
| 14 | -1 +1 -1 | 20 | 1000 | 1.8 | 3 |
| 15 | -1 +1 -2 | 20 | 1000 | 1.08 | 5 |
| 16 | 0 -1 +2 | 50 | 500 | 10.8 | 0.5 |
| 17 | 0 -1 +1 | 50 | 500 | 5.4 | 1 |
| 18 | 0 -1 0 | 50 | 500 | 2.7 | 2 |
| 19 | 0 -1 -1 | 50 | 500 | 1.8 | 3 |
| 20 | 0 -1 -2 | 50 | 500 | 1.08 | 5 |
| 21 | 0 0 +2 | 50 | 750 | 10.8 | 0.5 |
| 22 | 0 0 +1 | 50 | 750 | 5.4 | 1 |
| 23 | 0 0 0 | 50 | 750 | 2.7 | 2 |
| 24 | 0 0 -1 | 50 | 750 | 1.8 | 3 |
| 25 | 0 0 -2 | 50 | 750 | 1.08 | 5 |
| 26 | 0 +1 +2 | 50 | 1000 | 10.8 | 0.5 |
| 27 | 0 +1 +1 | 50 | 1000 | 5.4 | 1 |
| 28 | 0 +1 0 | 50 | 1000 | 2.7 | 2 |
| 29 | 0 +1 -1 | 50 | 1000 | 1.8 | 3 |
| 30 | 0 +1 -2 | 50 | 1000 | 1.08 | 5 |
| 31 | +1 -1 +2 | 50 | 500 | 10.8 | 0.5 |
| 32 | +1 -1 +1 | 50 | 500 | 5.4 | 1 |
| 33 | +1 -1 0 | 50 | 500 | 2.7 | 2 |
| 34 | +1 -1 -1 | 50 | 500 | 1.8 | 3 |
| 35 | +1 -1 -2 | 50 | 500 | 1.08 | 5 |
| 36 | +1 0 +2 | 50 | 750 | 10.8 | 0.5 |
| 37 | +1 0 +1 | 50 | 750 | 5.4 | 1 |
| 38 | +1 0 0 | 50 | 750 | 2.7 | 2 |
| 39 | +1 0 -1 | 50 | 750 | 1.8 | 3 |
| 40 | +1 0 -2 | 50 | 750 | 1.08 | 5 |
| 41 | +1 +1 +2 | 50 | 1000 | 10.8 | 0.5 |
| 42 | +1 +1 +1 | 50 | 1000 | 5.4 | 1 |
| 43 | +1 +1 0 | 50 | 1000 | 2.7 | 2 |
| 44 | +1 +1 -1 | 50 | 1000 | 1.8 | 3 |
| 45 | +1 +1 -2 | 50 | 1000 | 1.08 | 5 |

**Table S2** Experimental table comparison of hot-air drying

| **Experiment order** | **Temperature (°C)** | **Exposure time (s)** | **Velocity (m/min)** |
| --- | --- | --- | --- |
| 1 | 100 | 60 | 2.5 |
| 2 | 100 | 30 | 5 |
| 3 | 100 | 15 | 10 |

Table S3 lists the ANOVA results. The analysis was carried out using MINITAB commercial software. The transmittance results of all samples show the wavelength variations. Thus, the average value was calculated and analysed at an effective region of 800–1100 nm wavelengths. The input sources are denoted as A, B, and C representing distance, power, and exposure time, respectively. The interaction effect of ABC is very low; thus, it is pooled in the analysis. The F-value indicates the effect and significance of each factor. A large F-value is more significant than the other factors. In the NP case, the A value is the most significant factor for transmittance; the second most significant factor is the interaction effect between A and C. In the flake case, (B) is most critical factor, and the interaction effects are AB and BC. This is clearly shown as a normalised graph in Figure S4.

As shown in Figure S4(a), the NP case shows a minimal effect under the strongest sintering conditions (20-mm distance and 1,000-W power), caused by the reflectivity increase in the printed surface. In contrast, the flake shows a clearly sloping plot as a sintering tendency, as shown in Figure S4(e).

The interferometer-measured layer thicknesses and morphologies of the Ag NP and Ag flake are shown in Figures S5(a) and (b). The roughness values as calculated energy densities and thicknesses are shown as contour plots in Figures S5(c) and (d). Evidently, the relationship between the energy density and roughness value shows no tendency in the Ag NP. We presume that the colour change in the microscope image could have represented the change in the material properties in the binder or Ag materials. Moreover, tendency between the thickness and roughness on the Ag flake is apparent. The high roughness values are due to the large thickness value.

**Table S3** ANOVA of transmittance versus distance, power, and sintering time (exposure time) of the NP and flake paste

| **Paste** | **Source** | **DF** | **Seq SS** | **Adj SS** | **Adj MS** | **F** |
| --- | --- | --- | --- | --- | --- | --- |
| NP | A | 2 | 566.5 | 566.5 | 283.2 | 1.16 |
|  | B | 2 | 55.3 | 55.3 | 27.7 | 0.11 |
|  | C | 1 | 1 | 2.4 | 2.4 | 0.01 |
|  | AB | 4 | 247.7 | 247.7 | 61.9 | 0.25 |
|  | AC | 2 | 541.6 | 541.6 | 270.8 | 1.11 |
|  | BC | 2 | 206.6 | 206.6 | 103.3 | 0.42 |
|  | Error | 4 | 978.8 | 978.8 | 244.7 |  |
|  | Total | 17 | 2,598.8 |  |  |  |
|  | S = 15.6427; R^2^ = 62.34% | | | | | |
| **Paste** | **Source** | **DF** | **Seq SS** | **Adj SS** | **Adj MS** | **F** |
| Flake | A | 2 | 29.38 | 29.38 | 14.69 | 0.34 |
|  | B | 2 | 125.50 | 125.50 | 62.75 | 1.44 |
|  | C | 1 | 3.01 | 3.01 | 3.01 | 0.07 |
|  | AB | 4 | 53.19 | 53.19 | 13.30 | 13.3 |
|  | AC | 2 | 53.48 | 53.48 | 26.74 | 0.61 |
|  | BC | 2 | 715.56 | 715.56 | 357.78 | 8.18 |
|  | Error | 4 | 174.86 | 174.86 | 357.78 |  |
|  | Total | 17 | 1,154.98 |  |  |  |
|  | S = 6.61167; R^2^ = 84.86% | | | | | |

**Figure S4.** (a) Normalised primary effects of the experimental factor on the transmittance value. (b)–(d) Contour plot of the NP case. (e) Main effect on the (f)–(h) flake cases and their contour plots.

**Figure S5.** Interferometer-measured 3-D profile of gravure-printed and sintered (a) Ag NP and (b) Ag flake. Contour plot of the roughness value as the calculated relative energy density and thickness of (c) Ag NP and (d) Ag flake.

Figure S6 shows the primary-effect and contour plots of the sheet resistance with the change in the parameters. The NP pattern shows a clear primary effect of each parameter; however, the 20- and 50-mm-distance cases are similar, as shown in Figure S6(a). On the other hand, the flake pattern shows a clear and constant result with the change in each parameter. The contour plot represents the optimum value of the sheet resistance. The NP result shows the high slope (colour change) compared with the flake case. The lowest value of the NP case can be obtained at the nearest distance [Figure S6(b)] and longest exposure time [Figure S6(d)]. However, the lamp power does not have a significant effect compared with the other factors. In the flake case, the distance effect relatively decreases compared with the NP case [Figures S6(f) and (g)], and the power effect increases, which is shown as a symmetric shape in Figure S6(h).

**Figure S6.** (a) Normalised primary effects of the experimental factor to sheet resistance value. (b)–(d) Contour plot of the NP case and (e) primary effect on the (f)–(h) flake cases and their contour plots.

**Table S4** Comparison of performance with other studies

| **Sintering method** | **Before** | **After** | **Improve-ment (%)** | **Note** |
| --- | --- | --- | --- | --- |
|  | **Ω/□** | |  |  |
| This work | 424 | 0.288 | 147,222 | 1000-W power and 10.8-s sintering time |
| Electrical^1^ | 1,000 | 0.12 | 833,333 | 50-W power and 25-mm/s speed (0.3-s sintering time) |
| IR^2^ | 2.2 | 0.26 | 846 | 2,600 °C temperature and 1.25-s sintering time |
| Laser^3^ | 4,000 | 1 | 400,000 | 200-mW power and 10-s-sintering time |
| IPL^4^ | 271 | 6.83 | 3,968 | 2.8-kV voltage and 1-m/min web speed (2.4-s sintering time) |
| **Sintering method** | **Before** | **After** | **Improve**  **-ment (%)** | **Note** |
|  | **μΩ·cm** | |  |  |
| Thermal^5^ | 150 | 10 | 1,500 | 400-°C temperature and 20-min sintering time |
| Plasma^6^ | 10,000 | 3.98 | 251,256 | 300-W power and 60-min sintering time |
| UV^7^ | 25 | 3.7 | 675 | 70-mW/cm^2^ power and 30-min sintering time |
| Microwave^8^ | 2,000 | 2 | 100,000 | 2.45-GHz frequency, 1-W power, and 1-s sintering time |

**References (Supplementary Section)**

1. Allen M, Alastalo A, Suhonen M, Mattila T, Leppäniemi J, Seppä H. Contactless electrical sintering of silver nanoparticles on flexible substrates. *Microwave Theory and Techniques, IEEE Transactions* on **59** 1419-1429 (2011).
2. Sowade E, Kang H, Mitra KY, Weiß OJ, Weber J, Baumann RR. Roll-to-roll infrared (IR) drying and sintering of an inkjet-printed silver nanoparticle ink within 1 second. *Journal of Materials Chemistry C* (2015).
3. Yeo J, et al. Flexible supercapacitor fabrication by room temperature rapid laser processing of roll-to-roll printed metal nanoparticle ink for wearable electronics application. *Journal of Power Sources* **246**, 562-568 (2014).
4. Hösel M, Krebs FC. Large-scale roll-to-roll photonic sintering of flexo printed silver nanoparticle electrodes. *Journal of Materials Chemistry* **22**, 15683-15688 (2012).
5. Park J, Nguyen HA, Park S, Lee J, Kim B, Lee D. Roll-to-roll gravure printed silver patterns to guarantee printability and functionality for mass production*. Current Applied Physics* **15**, 367-376 (2015).
6. Wolf FM, Perelaer J, Stumpf S, Bollen D, Kriebel F, Schubert US. Rapid low-pressure plasma sintering of inkjet-printed silver nanoparticles for RFID antennas. *Journal of Materials Research* **28**, 1254-1261 (2013).
7. Jahn SF, et al. Inkjet printing of conductive silver patterns by using the first aqueous particle-free MOD ink without additional stabilizing ligands. *Chemistry of Materials* **22**, 3067-3071 (2010).
8. Perelaer J, Klokkenburg M, Hendriks CE, Schubert US. Microwave flash sintering of inkjet-printed silver tracks on polymer substrates. *Advanced Materials* **11**, 4830 (2009).
